# Efficacy of Keyhole Approach to Carpal Tunnel Syndrome under Ambulatory Strategy

**DOI:** 10.1155/2017/3549291

**Published:** 2017-04-06

**Authors:** Rodrigo Ramos-Zúñiga, César J. García-Mercado, Iván Segura-Durán, Luis A. Zepeda-Gutiérrez

**Affiliations:** Translational Institute of Neuroscience, Department of Neuroscience, CUCS, University of Guadalajara, Guadalajara, JAL, Mexico

## Abstract

The carpal tunnel syndrome is one of the most common entrapment neuropathies found in humans. Currently, the gold standard is surgical treatment using different modalities. The minimally invasive strategy with high resolution capacity and less morbidity is still a challenge.* Methods*. Prospective nonrandomised clinical trial in which a minimally invasive microsurgical approach was used following the keyhole principle in 55 consecutive patients and 65 hands under local anesthesia and ambulatory strategy. They were evaluated with stringent inclusion criteria with the Levine severity and functional status scale and with a 2-year follow-up.* Results*. 90% showed immediate improvement dropping to grades 1-2 in all items of the scale referring to pain and numbness. 97% reported improvement, as of the first month, and 3% reported persistence of symptoms, although at a lesser degree and with no functional limitation. No incidents were identified during the procedure and 98% of patients were discharged within an hour after the surgical procedure.* Conclusions*. The microsurgical approach described following the keyhole principle is a treatment option that, under local anesthesia and ambulatory management, may represent an alternative strategy of an effective treatment reducing the morbidity. This trial is registered with Clinical Trials Protocol Identifier NCT03062722.

## 1. Introduction 

Carpal tunnel syndrome (CTS) is the most common form of entrapment neuropathy in the upper limb and it is estimated to occur in 3.8% of the general population. The age group in which it is commonly found is 30 to 60 years, with females being affected three times more than men. The overall prevalence is 3.7% and it is estimated that more than 500.000 patients are subjected to carpal tunnel release (CTR) every year. CTS has been rated second among the main diagnoses in work absences and estimated economic cost. It has been considered that one out of five individuals who complain of pain, numbness, and tingling in hands could have CTS [1–3].

The main feature in carpal tunnel syndrome is the compression of the median nerve in the wrist due to a tighter free space in the carpal canal or tunnel and consequently there is greater pressure that may lead to reduced functional capacity, loss of dexterity, hand numbness, and loss of muscle mass. A collegiate classification proposes finding three or more of the following signs and symptoms to establish the diagnosis: (1) paresthesia along the territory of median nerve, (2) nocturnal paresthesia, (3) thenar atrophy, (4) positive Tinel test, (5) positive Phalen test, and (6) decreased sensitivity.

In most cases the origin is considered idiopathic, although it has been associated with other causes as inflammatory arthropathies such as rheumatoid arthritis. Trauma, diabetes, acromegaly, hypothyroidism, and pregnancy are described also [4, 5].

According to biomechanical and histological results, the most typical histologic finding is inflammatory fibrosis and thickening of the subsynovial connective tissue. Nowadays, the preferred surgical treatment for definitive resolution of CTS is undebatable, but the current challenge is to evaluate which is the most efficacious and less invasive strategy to resolve the entrapment [6, 7]. This overview should consider not only the surgical strategy and its described variants, wide open, mini-open, endoscopic, or percutaneous techniques [8–16], but also the anesthetic procedure in different ways [17–22], hospital stay, functional recovery, and potential recurrences as part of the analysis [23, 24]. In this study we evaluate the efficacy of the keyhole strategy applied to the microsurgical approach of the carpal tunnel syndrome.

## 2. Methods 

This was a prospective nonrandomised clinical study to analyze 55 consecutive series of patients with carpal tunnel syndrome treated with mini-open minimally invasive approach in 65 hands, using local anesthesia without tourniquet and in an ambulatory setting.

The inclusion criteria were patients with confirmed diagnosis who had neurologic exam, electromyography, and electroneuronogram with nerve conduction evidence of severe CTS in agreement with distal motor latency > 6 ms, decreased sensory conduction and decreased amplitude rate, and cervical spine X-rays showing no structural disturbances and with at least 3 months of persistent pain refractory to medical management and physical therapy. Patients with a history of direct trauma or orthopedic lesions in the carpal region and endocrine and/or metabolic disturbances (hypothyroidism, diabetes) and those that had previous local administration of steroids were excluded.

### 2.1. Surgical Technique

**Figure 1:**
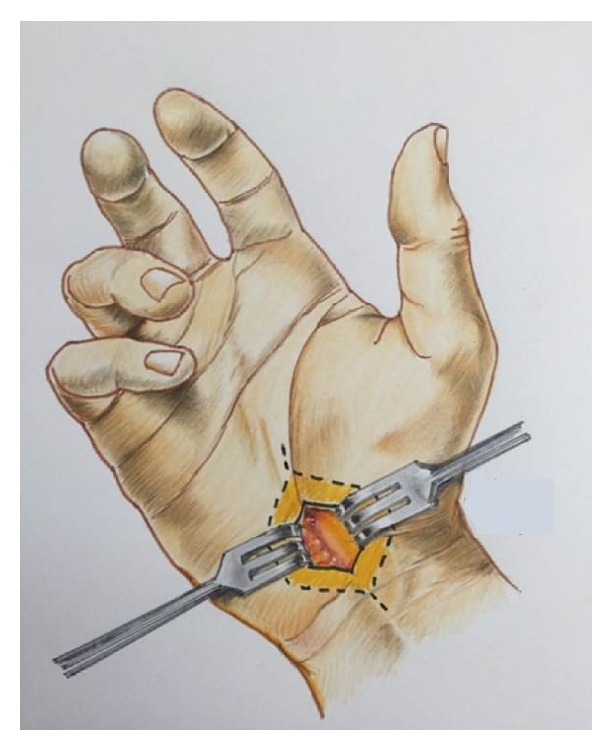
Artistic draw to show the key hole principle applied to the carpal tunnel microsurgical release.

The surgical procedure was a direct microsurgical approach with a 1.5 cm incision in the thenar sulcus, under local anesthesia (3 cc, 2% lidocaine) administered with an insulin needle. The keyhole approach applied to this anatomical region is based on a 1.5 cm skin incision from where the 0.5 cm dissection is completed in the subcutaneous plane in the side borders and 1 cm in the distal and proximal borders. Thus, the subcutaneous phase of the dissection is completed with separation of the carpal ligament and resection of its borders. Once the transverse fibers of the flexor retinaculum are open medially under surgical loupes and headlight, the perineural microadhesions of the median nerve are resected and 3 mm of the free borders of the carpal fibers, found on the nerve, is removed and coagulated with bipolar gently to avoid fibrosis. The wound is checked for hemostasis and closed in apposition with Vicryl 3-0 and a single subdermal 3-0 Nylon stitch (Figure 1).

### 2.2. Follow-Up

After one hour of the procedure, the patient was discharged with analgesics, antibiotic, and an external splint for the wrist support. Physical therapy for passive and fine motor skills was recommended three weeks after surgery in every case.

The patients were followed up postoperatively for 10 days, 1 month, 6 months, 1 year, and 2 years with Levine's scale to evaluate the severity of symptoms [25], as well as to evaluate the patients' functional status in daily activities.

## 3. Results 

All the consecutive patients operated on were females because we do not see any case of male patients in this study, of which 70% had bilateral symptoms with predominance of the dominant hand. All the patients had at least 3 months of pain and were considered refractory to conservative treatment and met the inclusion criteria.

95% of cases had a preoperative clinical course between 3 and 6 months. 5% had an average one-year course, diagnosed as nerve root syndrome.

All patients required a second administration of local anesthetic before closing the skin. Exclusively in teen patients, a complementary 5 mg intramuscular injection of Midazolam was necessary during the surgery, for a better control of anxiety.

In 10% of cases, both hands were operated on, with two months' interval.

Likewise, 35% of patients improved their contralateral symptoms after surgery in the first hand, reason for which an alternate surgery of the contralateral hand was ruled out.

Three cases were identified as having associated cervical nerve root symptoms during follow-up and had ACDF (Anterior Cervical Discectomy and Fusion) surgery of C5-C6 a year after.

According to the Levine Severity Scale [25], all patients were classified in the most severe grade: 4-5 of the 11 items. 90% showed immediate improvement as they moved to grades 1-2 in every item of the scale on reference to pain and numbness (10 days). 7% presented the described improvement from the first month and 3% reported persistence of symptoms although at a lesser degree and without functional limitation. The most chronic and severe cases in electrophysiology studies were included in this 3%, even the case with an associated neuropsychiatric anxiety disorder.

For long-term follow-up, the functional status scale was used [25], confirming the previous results, concerning daily activities.

**Figure 2:**
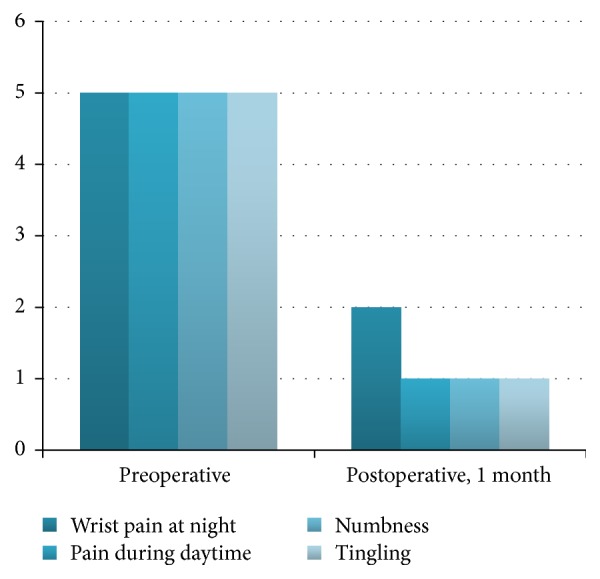
Symptom severity scale (Levine).

**Figure 3:**
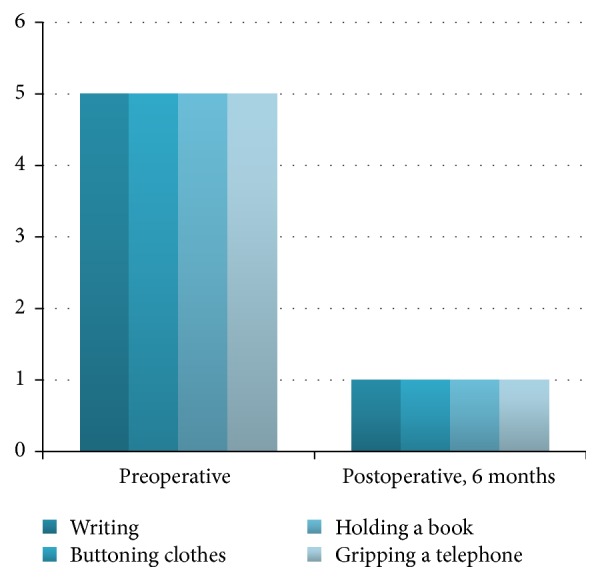
Functional status scale (Levine).

85% of the patients were classified grade 1 category at 6 months' evaluation (no difficulty) and 12% were grade 2 (mild difficulty). At the one-year evaluation, 97% were reported grade 1 in the clinical evaluation (Table 1 and Figures 2 and 3).

## 4. Discussion 

Treatment for carpal tunnel syndrome offers different treatment options, including conservative management, physical therapy, use of splints, TENS (Transcutaneous Electrical Nerve Stimulation), and drug treatment with different modalities, as well as directly resorting to application of steroids. However, in many cases, conservative treatment only involves a relative improvement and palliative treatment. The use of steroids may be effective for transiently reducing inflammation and edema in the synovial membrane and tendons, but they also have a potentially harmful effect on tissue function by reducing collagen and synthesis of proteoglycans, which reduces the mechanical resistance of the tendon and leads to greater degeneration [1, 2].

When conservative treatment is considered insufficient due to persistent pain and functional results, a simple decompression of the median nerve is indicated by section of the transverse carpal ligament, which is the most successful surgical treatment. The open surgical approach to the carpal tunnel has been the gold standard approach, and it is considered to have good outcomes in 75% of the operated patients.

It has been proven that surgical treatment of the CTS is the best option to improve the disease long-term symptoms, since most of the patient series report 81.7% improvement of sensory function, functional status, and subjective hand symptoms. Complete recovery is found in 99% of patients with mild conduction disturbances and 94% of those with moderate anomalies in most of the reported series. The combination of surgical treatment and rehabilitation in the early postoperative period gives the best outcomes.

A survey report from the American Association for Hand Surgery from 2012 found that most surgeons use local anesthesia, 33.4% are in favor of a standard open incision, while 45.5% favor a mini-incision, and 19.5% prefer an endoscopic release. Among the surgeons who have the largest clinical practice, the surgical method of choice is a “mini-open” procedure. Injection of steroids to alleviate symptoms in CTS is regularly used by 63.2% of surgeons in clinical practice, and endoscopic release is the most used surgical approach by 17.2% of plastic surgeons and 20% of orthopedic surgeons [3, 4].

From the neurosurgical perspective, functional preservation of the median nerve is crucial, without compromising the biomechanical function of the carpal region and avoiding recurrence associated with fibrosis.

Although the current proposal does not intend to compare the efficacy of this procedure with others, it does support that it is possible to systematize a minimally invasive method in the surgical procedure of a peripheral nerve under the keyhole principle, using ambulatory criteria and with the least possible morbidity involved in the regional anesthetic procedure, with good tolerance and comfort for the patient during the operation and in the immediate postoperative period.

In addition, it is not necessary to admit patients into the hospital floor since the procedure is ambulatory and can be admitted directly to the operation room. In most cases patients can be discharged after one hour of the surgery.

We did not use a tourniquet in any of our patients [16], which in our view is an unnecessary traumatic event; and the use of lidocaine as a local anesthetic without epinephrine does not compromise the microcirculation of the perineurium, requiring only additional application prior to closure.

A key point concerning the benefits of the microsurgical resection under the keyhole principle is that since it is a small incision, the risk of keloid scars in the area is reduced, and the dissection can be complemented at the distal and proximal level of the flexor retinaculum transverse fibers in the subcutaneous plane [26].

In addition, a delicate resection of the free edges of the ligament can be performed rather than simply dissecting, reducing the risk of perineurium's intrinsic fibrosis.

It is noteworthy that the published literature additionally reports that improvement of the contralateral hand was found in 74–84% of cases, in objective and subjective evaluations with stable beneficial effects at 180 days [27]. This report showed that gender, age, professional status, duration of preoperative symptoms, and electrophysiological severity were not predictive. This condition established the possibility of neuroplasticity involvement in the impact of local regeneration on one hand, as well as at cortical levels in the centers associated with sensitivity and pain circuits. This proposal is related to a reorganization of the somatosensory cortex in experimental studies as a sensory recovery process in common regions for both hands as a theory [28].

It is essential for the practicing neurosurgeon to identify this condition in the differential diagnosis with cervical root syndrome, to establish the correct diagnosis and rationally propose the most appropriate surgical procedure. This is relevant per the reported experience, since 5% of cases were diagnosed as root syndrome and other cases not included in this series had ACDF surgery with no clinical improvement, while the peripheral nerve problem was still unresolved.

Carpal tunnel microsurgery with local anesthesia has become a good treatment option because it is fast, safe, and effective. The ambulatory strategy allows reducing the morbidity involved in more invasive anesthetic procedures, hospital stay, and the costs for the procedure. Furthermore, the patient can return sooner to his daily activities [23, 24]. Related to other reports this proposal can be available as a strategy for specific cases of CPS [29, 30].  Table 2.

## 5. Conclusion 

The minimally invasive procedure following the keyhole principle can be applied to carpal tunnel syndrome, under local anesthesia, without sedation in the majority of cases and ambulatory scheduled surgical procedure with successful outcomes and problem resolution in agreement with most common surgical techniques. This strategy is not intended to be compared to other techniques in terms of efficacy, but in our specific context it is a good option to resolve the mechanical compression. Additionally, the risks involved in major invasive anesthetic procedures are reduced, as well as costs.

## Figures and Tables

**Table 1:** Evaluation of cases at long term with the functional status scale in daily activities.

Preoperative inclusion criteria	Symptom severity scale	Microsurgical carpal tunnel approach under local anesthesia	Discharge, 1 H after procedure	Complications	Functional status scale at 6 months	Functional status at 1 year
100%	Grades 4-5 100%	100%	98%	No	Grade 1 85%	Grade 1 97%

**Table 2:** Comparative results in summary, of carpal tunnel release in the literature.

Author	Cases	Surgery	Outcome
Guo et al. 2015 [9]	20	Percutaneous nonscalpel Local anesthesia, ambulatory surgery	Improvement; effective, low cost, reduced recovery time

Michelotti et al. 2014 [13]	25	Open and endoscopic (self-control)	Improvement; no differences; more overall satisfaction in endoscopic surgery

Aslani et al. 2014 [7]	48	Open and endoscopic surgery	Clinical improvement, image of carpal canal in postoperative follow-up

Leblanc et al. 2007 [23]	Survey or surgeons	Open release Local anesthesia Operating room versus ambulatory surgery	Improvement; low cost in ambulatory setting (37%)

Means Jr. et al. 2014 [12]	91	Single portal endoscopic surgery	Long-term efficacy, low recurrence

Murthy et al. 2015 [15]	134	Mini-open versus extensile release	Improvement; no differences between groups

Davison et al. 2013 [19]	200	Open and endoscopic Local anesthesia with or without sedation	Sedated patients spent more time in hospital and more preoperative testing

Our study 2016	55	Mini-open key hole; local anesthesia, ambulatory setting	Effective; preoperative nerve conduction studies, symptom severity scale (Levine), functional status, improvement in VAS, ambulatory surgery, low cost

## Abbreviations

CTS: Carpal tunnel syndrome
CTR: Carpal tunnel release
ACDF: Anterior Cervical Discectomy and Fusion
TENS: Transcutaneous Electrical Nerve Stimulation.
